# Injectable supramolecular polymer–nanoparticle hydrogels enhance human mesenchymal stem cell delivery

**DOI:** 10.1002/btm2.10147

**Published:** 2019-10-22

**Authors:** Abigail K. Grosskopf, Gillie A. Roth, Anton A. A. Smith, Emily C. Gale, Hector Lopez Hernandez, Eric A. Appel

**Affiliations:** ^1^ Department of Chemical Engineering Stanford University Stanford California; ^2^ Department of Bioengineering Stanford University Stanford California; ^3^ Department of Materials Science & Engineering Stanford University Stanford California; ^4^ Department of Biochemistry Stanford University Stanford California

**Keywords:** cell delivery, human mesenchymal stem cell, hydrogel, injectable

## Abstract

Stem cell therapies have emerged as promising treatments for injuries and diseases in regenerative medicine. Yet, delivering stem cells therapeutically can be complicated by invasive administration techniques, heterogeneity in the injection media, and/or poor cell retention at the injection site. Despite these issues, traditional administration protocols using bolus injections in a saline solution or surgical implants of cell‐laden hydrogels have highlighted the promise of cell administration as a treatment strategy. To address these limitations, we have designed an injectable polymer–nanoparticle (PNP) hydrogel platform exploiting multivalent, noncovalent interactions between modified biopolymers and biodegradable nanoparticles for encapsulation and delivery of human mesenchymal stem cells (hMSCs). hMSC‐based therapies have shown promise due to their broad differentiation capacities and production of therapeutic paracrine signaling molecules. In this work, the fundamental hydrogel mechanical properties that enhance hMSC delivery processes are elucidated using basic *in vitro* models. Further, *in vivo* studies in immunocompetent mice reveal that PNP hydrogels enhance hMSC retention at the injection site and retain administered hMSCs locally for upwards of 2 weeks. Through both *in vitro* and *in vivo* experiments, we demonstrate a novel scalable, synthetic, and biodegradable hydrogel system that overcomes current limitations and enables effective cell delivery.

## 1 INTRODUCTION

Stem cells delivered to native tissues aid in tissue regeneration through integration and act as sources of therapeutic paracrine factors. Notably, research into human mesenchymal stem cells (hMSCs) has taken off due to hMSC's broad differentiation capacity into osteogenic, chondrogenic and adipogenic lineages.^1^, ^2^ Compared to embryonic stem cells and induced pluripotent stem cells, hMSCs are broadly accessible from bone marrow, do not pose ethical concerns, and exhibit low teratoma formation and immunogenicity.^3^ hMSCs, sometimes even referred to as human medicinal signaling cells,^4^ also produce high levels of complex therapeutic paracrine molecules to aid in wound healing and recruit other cells throughout the body.^5^ Due to these widespread capabilities, hMSCs are the most common cell type used in clinical cell therapy applications to treat myocardial infarctions, osteochondral defects, spinal cord injuries and graft versus host disease.^3^, ^6^, ^7^ For hMSC therapies to be most effective they require high hMSC engraftment and localization after injection. Additionally, the complex interactions between hMSCs and the inflammatory immune response limit the efficacy of hMSCs' therapeutic capabilities.^3^

There are many challenges in the local delivery of stem cell therapeutics.^8^ Cells encounter gravitational forces and strong bodily pressures diminishing cell retention at the injection site.^9^, ^10^ Injected cells must also interact with the immune system. The leading methods for local delivery involve hydrogels, which are biomimetic water swollen polymer networks that are capable of suspending cells in 3D.^8^, ^11^, ^12^ Hydrogels can increase local cell retention and engraftment at the injection site compared to traditional liquid injections or infusion by acting as local niches to physically hold cells in place.^13^ Additionally hydrogels can act as immunomodulatory protective barriers against infiltrating immune cells.^14^ The benefits of encapsulating hMSCs in hydrogels have been shown in multiple clinical studies targeting an array of indications.^10^, ^15^, ^16^

Yet, crosslinked hydrogels containing cells often require invasive implantation procedures.^17^ The development of shear‐thinning hydrogels has enabled quicker, less invasive administration procedures by injection through a needle.^16^, ^18^, ^19^, ^20^, ^21^, ^22^, ^23^ Injectable hydrogels enhance cell viability during the injection process compared to liquid vehicles by alleviating the mechanical forces cells experience when traveling through small diameter needles.^19^, ^23^ However, injectable shear‐thinning hydrogels are often formed through weak physical interactions and cannot persist long enough in the body to enhance cell retention compared to traditional liquid injections.^21^ To circumvent this challenge, researchers have developed stimuli responsive and dynamic hydrogel materials that change properties *in situ* after injection.^12^, ^13^, ^20^, ^24^, ^25^, ^26^ Hydrogels based on polymers exhibiting lower critical solution temperature (LCST) behavior that are injectable liquids at room temperature but gel in situ at physiological temperature after injection have been developed^13^, ^20^, ^24^; however, these “thermogelling” hydrogels can be difficult to handle due to their inherent temperature sensitivity, often involve harsh complex chemistries, and often suffer from cells settling in the syringe prior to injection.^8^ Moreover, many LCST‐based hydrogels rely on high molecular weight polymers, including poly(N‐isopropylacrylamide) (PNIPAM), which are not biodegradable.^27^ Further, many other injectable hydrogel platforms are composed of natural materials such as collagen and alginate, and as such are subject to batch‐to‐batch variability and lack mechanical tunability, or are composed of expensive and poorly scalable protein‐engineered materials.^8^ It is important to note that while numerous reported studies have focused on developing hydrogels for enhanced delivery and retention of various cell types, engineering the material properties of hydrogels for robust hMSC delivery and retention has only rarely been investigated.

Here we present the use of a novel injectable shear‐thinning supramolecular polymer–nanoparticle (PNP) hydrogel made from a mixture of dodecyl‐modified hydroxypropylmethylcellulose (HPMC_12_) and biodegradable nanoparticles comprising poly(ethylene glycol)‐*block*‐poly(lactic acid) (PEG–PLA NPs) to enhance the local retention of hMSCs after injection. Dynamic PNP interactions have previously been shown to create injectable hydrogels exhibiting long‐term delivery of therapeutics (small molecules and biologics) and enhance cell viability during injection.^28^, ^29^, ^30^ These materials are injectable and biodegradable. The strong supramolecular interactions between the functional biopolymers and the NPs that give rise to the hydrogel structure yield *in vivo* depots capable of persisting for long timescales in the body.^31^ In this article, we first identify the fundamental mechanical properties of hydrogels needed for the entire cell delivery process with *in vitro* experiments. We then demonstrate enhanced hMSC retention with PNP hydrogels *in vivo* compared to traditional liquid injections.

## 2 RESULTS AND DISCUSSION

### 2.1 Design of PNP hydrogels for cell delivery

Cellulose derivatives are widely available, inexpensive, and biocompatible high molecular weight polymers commonly used as viscosifiers in pharmaceutical formulations,^32^ cosmetics,^33^ and a broad array of industrial applications.^34^ In our hydrogel system, hydroxypropylmethylcellulose (HPMC) was modified with hydrophobic lipid dodecyl chains (C_12_) using isocyanate coupling chemistry.^29^ PEG–PLA NPs with a diameter of ~30 nm were prepared using nanoprecipitation techniques,^29^, ^35^ yielding core‐shell NPs with a hydrophilic PEG‐based corona and a hydrophobic PLA‐based core. To promote cellular adhesion and viability, the cell adhesion motif arginine‐glycine‐aspartic acid (RGD) was attached to the hydrophilic end of the PEG–PLA copolymer through a copper‐catalyzed “click” reaction prior to nanoprecipitation. A 50:50 physical mixture of RGD‐functionalized PEG–PLA polymer (RGD‐PEG–PLA) and unmodified PEG–PLA polymer was used to create RGD‐functionalized PEG–PLA NPs.

### 2.2 PNP hydrogel formation and mechanical properties

**Figure 1:**
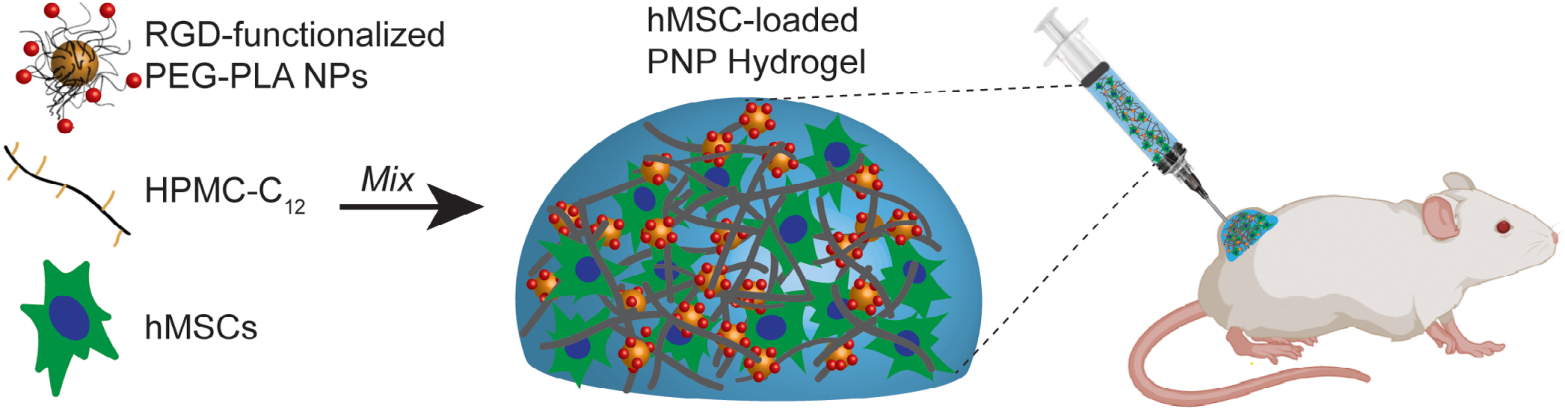
Polymer–nanoparticle (PNP) hydrogels for cell encapsulation and delivery. Aqueous solutions of RGD‐functionalized PEG–PLA NPs, HPMC‐C_12_, and hMSCs are mixed to form PNP hydrogels. These hMSC‐loaded hydrogels are easily injected through high‐gauge needles and can be used to enhance cell viability during injection and local retention at the injection site in immunocompetent mice

PNP hydrogels are formed by mixing aqueous solutions of the HPMC‐C_12_ polymer and RGD‐functionalized PEG–PLA NPs (Figure 1). Cells are easily suspended in the NP aqueous phase before mixing the NP and polymer solutions. When these components are mixed, dynamic multivalent interactions between the hydrophobically‐modified HPMC polymers and the core of the PEG–PLA NPs cause physical crosslinking and hydrogel formation. The mechanical properties of the resulting hydrogels depend on the concentration of polymer and nanoparticles present in the hydrogel. We generated three varying formulations of the PNP hydrogels: 1:1 PNP, 1:5 PNP, and 2:10 PNP, where the first number denotes the wt.% polymer and the second number denotes the wt.% NPs in the formulation.

**Figure 2:**
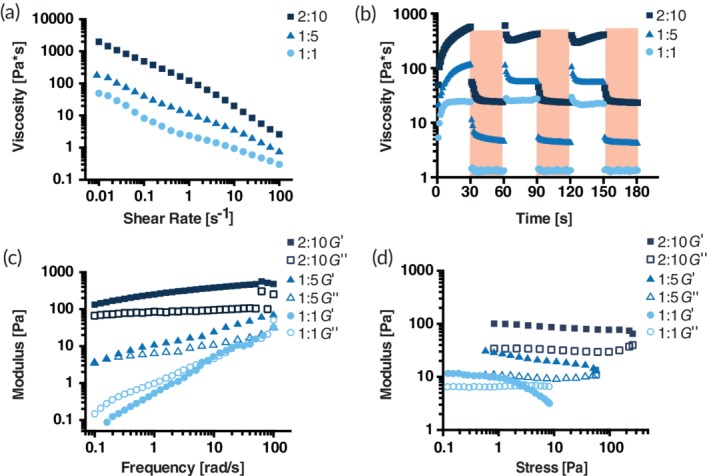
Rheological properties of three varying PNP formulations. PNP hydrogel formulations are denoted as polymer wt.%: NP wt.%. (a) Steady shear flow sweeps from low to high shear rate of PNP hydrogels. Viscosity as a function of shear rate characterizes shear‐thinning properties. (b) Viscosity as a function of oscillating shear rates between low (white background) 0.1 s^−1^ shear rates to high (red background) 10 s^−1^ shear rates demonstrating self‐healing properties of PNP hydrogels. Shear rates are imposed for 30 s each. (c) Elastic storage modulus *G*′ and viscous loss modulus *G*″ as a function of frequency at a constant 2 μNm torque (1.27 Pa) for various PNP hydrogels. (d) Amplitude sweeps at a constant frequency of 10 rad/s. Elastic storage modulus *G*′ and viscous loss modulus *G*″ of PNP hydrogels as a function of stress

Many hydrogel‐based stem cell therapies require surgical implantation due to the static nature of covalent crosslinks between polymers.^17^ These invasive administration techniques limit the scalability of the treatment and can cause undesirable side effects. The supramolecular PNP hydrogel platform we describe here exhibits shear‐thinning behavior enabling injection through small diameter needles. Shear‐thinning behavior was measured for all formulations using steady shear flow sweeps from low to high shear rates (Figure 2a). As the NP and polymer concentrations are increased, the viscosity of the material increases. All formulations see approximately a 3‐order of magnitude decrease in viscosity with increasing shear rates across the range tested. When these PNP hydrogels are injected and sheared through a needle, the supramolecular interactions between the polymer chains and the NPs are broken. After injection, the supramolecular interactions rapidly reform leading to complete self‐healing behavior. This behavior is ideal for delivery applications where facile delivery through injection and rapid formation of a strong and resilient material after injection are desirable. Self‐healing of the PNP hydrogel formulations was measured by imposing alternating intervals (30 s) of high (10 s^−1^) and low (0.1 s^−1^) shear rates (Figure 2b). Viscosity dropped quickly once high shear rates were applied, and completely and rapidly (<5 s) recovered once low shear rates were applied.

Viscoelastic properties were measured using frequency sweeps within the linear viscoelastic regime (torque = 2 μNm; Figure 2c). The inverse of the frequency at which *G*′ (elastic storage modulus) and *G*″ (viscous loss modulus) are equal describes a characteristic relaxation time for the hydrogel network. The higher the relaxation time, the longer it takes the material dissipate to stress and flow, indicating the material may exhibit longer residence upon implantation in the body. Our results demonstrate the tunability of the relaxation times through variations in the concentrations of polymer and NPs.

Another important material property of these hydrogels is the yield stress, which describes the stress required to initiate flow in a material. A higher yield stress corresponds to greater resilience and may lead to greater persistence in the body and greater cell retention. To determine the yield stress of our formulations, stress amplitude sweeps were performed at a constant angular frequency (*ω* = 10 rad/s; Figure 2d). The stress at which *G*′ and *G*″ cross over one another, indicating a switch from “solid‐like” to “liquid‐like” behavior, is often characterized as the yield stress.^36^ The yield stress may also be characterized through plotting stress as a function of shear rate (Figure S1). As the polymer and NP concentrations are increased in the hydrogel formulations, the yield stress increases accordingly. While it is hypothesized that both longer relaxation time and higher yield stress can lead to increased material residence in the body, many other complex factors affect hydrogel persistence *in vivo*.

### 2.3 PNP hydrogel mechanical properties dictate a uniform cell suspension

Heterogeneous injections arising from cell settling are a fundamental problem in cell delivery with traditional liquid injections. In an attempt to uniformly suspend the therapeutic cells in the liquid solution and avoid highly variable cell concentrations clinicians must vigorously mix the cell suspensions before injection. Adhesive cells tend to aggregate and adhere to the syringe and plunger, so many cells are lost in the process of loading the syringe and injecting. Hydrogels are an excellent medium to create homogenous cell suspensions and prevent cell aggregation on the syringe.^37^ A successful hydrogel for cell delivery must have mechanical properties that support the stress imposed by the cells due to gravity to prevent cell settling. The hydrogels must either have a high enough viscosity to slow settling over timescales relevant to injection (approximately 4 hr) or possess a yield stress higher than the stress imposed by the cells to completely stop settling.

**Figure 3:**
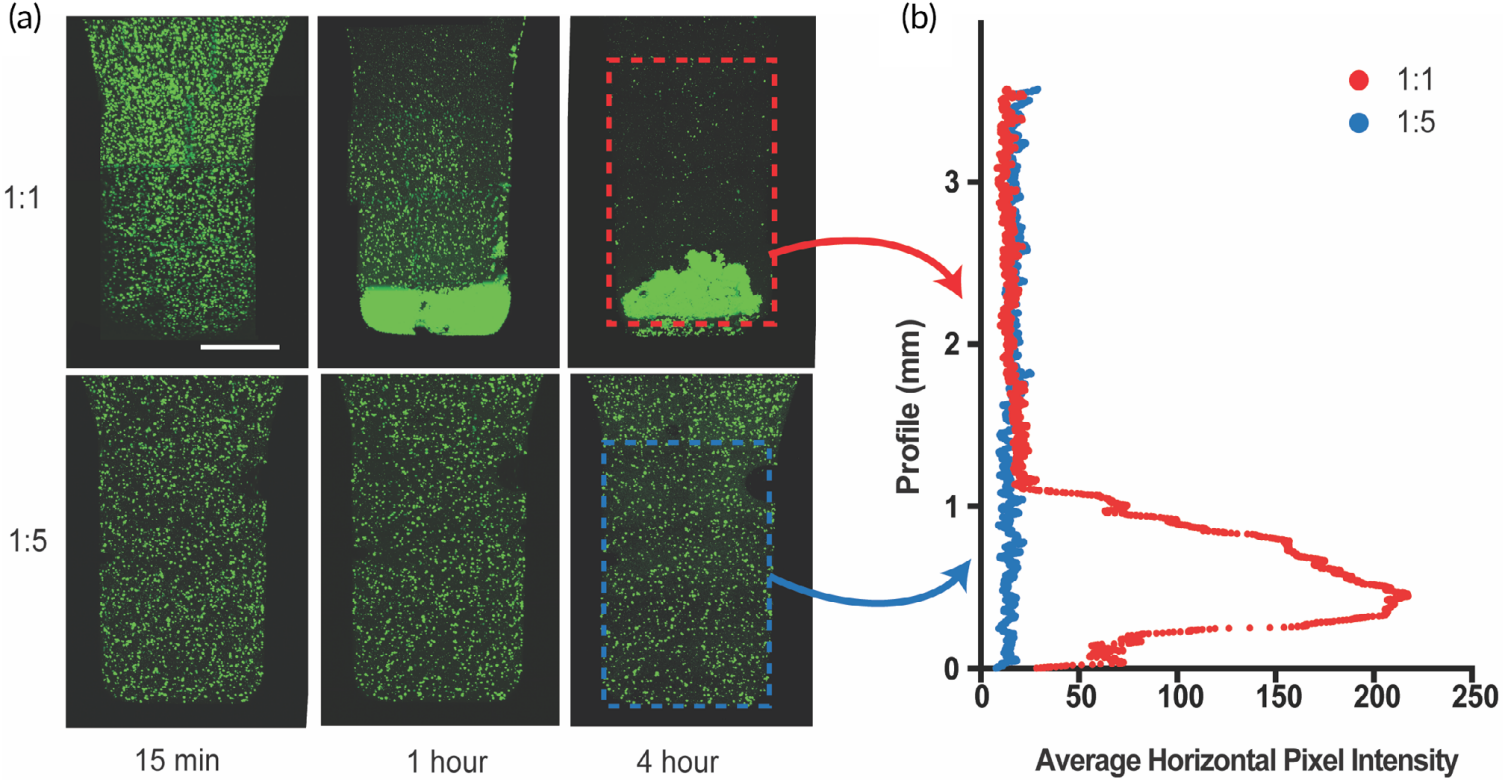
Cell encapsulation and settling experiments. (a) Cell settling maximum intensity images of calcein‐stained hMSCs encapsulated in 1:1 PNP hydrogel (top row) and 1:5 PNP hydrogel (bottom row) across 4 hr. Scale bar represents 1 mm. (b) Average horizontal pixel intensity of hMSCs along the vertical profile of the hydrogel

To assess whether PNP hydrogels can maintain uniform cell suspensions and prevent cell settling, hMSCs were labeled with calcein dye and encapsulated in PNP hydrogels (Figure 3). Gravity driven cell settling was imaged over the course of 4 hours. The 1:1 PNP formulation was not capable of supporting cells over this timescale, due to the lack of an appreciable yield stress. In contrast, the 1:5 PNP formulation supported cells throughout the entire 4 hr. The 1:5 PNP hydrogel possesses both a higher viscosity and a yield stress of approximately 60 Pa. These experiments suggest that hydrogel materials must reach critical threshold of mechanical properties to prevent cell settling over time.

### 2.4 hMSC cell viability in PNP hydrogels

**Figure 4:**
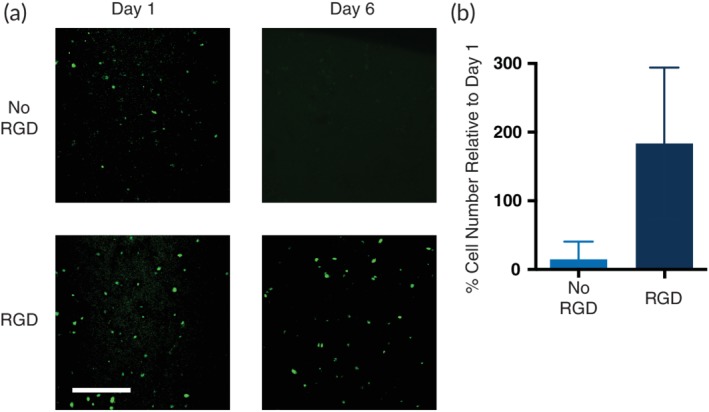
Cell viability studies in PNP hydrogels. (a) Representative images of viable hMSCs in 1:5 PNP hydrogels with and without the cell adhesive RGD motif attached to the PEG–PLA NPs. PNP hydrogels were calcein stained for 30 min prior to confocal imaging. Scale bar represents 100 μm. (b) Cell viability on Day 6 defined as number of fluorescent cells in the image relative to number of fluorescent cells on Day 1 (data shown as mean ± *SD*; *n* = 3)

Cell delivery materials for *in vivo* applications need to maintain viable cells for timescales long enough for the material to integrate with the body. Adhesive cells, including hMSCs, require integrin engagement to maintain viability. Many researchers incorporate natural biological materials such as collagen, gelatin, or matrigel into hydrogel materials to provide these necessary signals; however, natural substances suffer from batch‐to‐batch variability, introduce additional mixing steps into the fabrication process, and can sometimes cause long‐term immune effects. Alternatively, small and defined cell adhesive motifs can be synthetically introduced into materials to allow for cell adhesion.^38^, ^39^ Accordingly, we hypothesized that incorporation of RGD‐functionalized PEG–PLA NPs into PNP hydrogels would promote cell viability. To understand the effects of RGD incorporation into the PNP hydrogel structure on cell growth, hMSCs were cultured over the course of 6 days in 1:5 PNP hydrogels prepared with and without the RGD‐functionalized PEG–PLA NPs (Figure 4). These PNP hydrogels were prepared with a mixture of plain PEG–PLA NPs and RGD‐functionalized PEG–PLA NPs with a concentration of RGD in the hydrogel of 500 μM, which has been shown previously to be sufficient to support hMSC viability.^39^ In these experiments, hMSC viability was assessed through calcein staining on Days 1 and 6. The RGD motif enhanced cell viability over time and promoted hMSCs viability and expansion in the PNP hydrogel as an increase in viable cells was observed on Day 6 in the RGD‐functionalized PNP hydrogel. hMSCs were viable in PNP with no RGD sequence after Day 1, but viable hMSCs drastically decreased by Day 6.

### 2.5 PNP hydrogel mechanical properties control tissue integration

**Figure 5:**
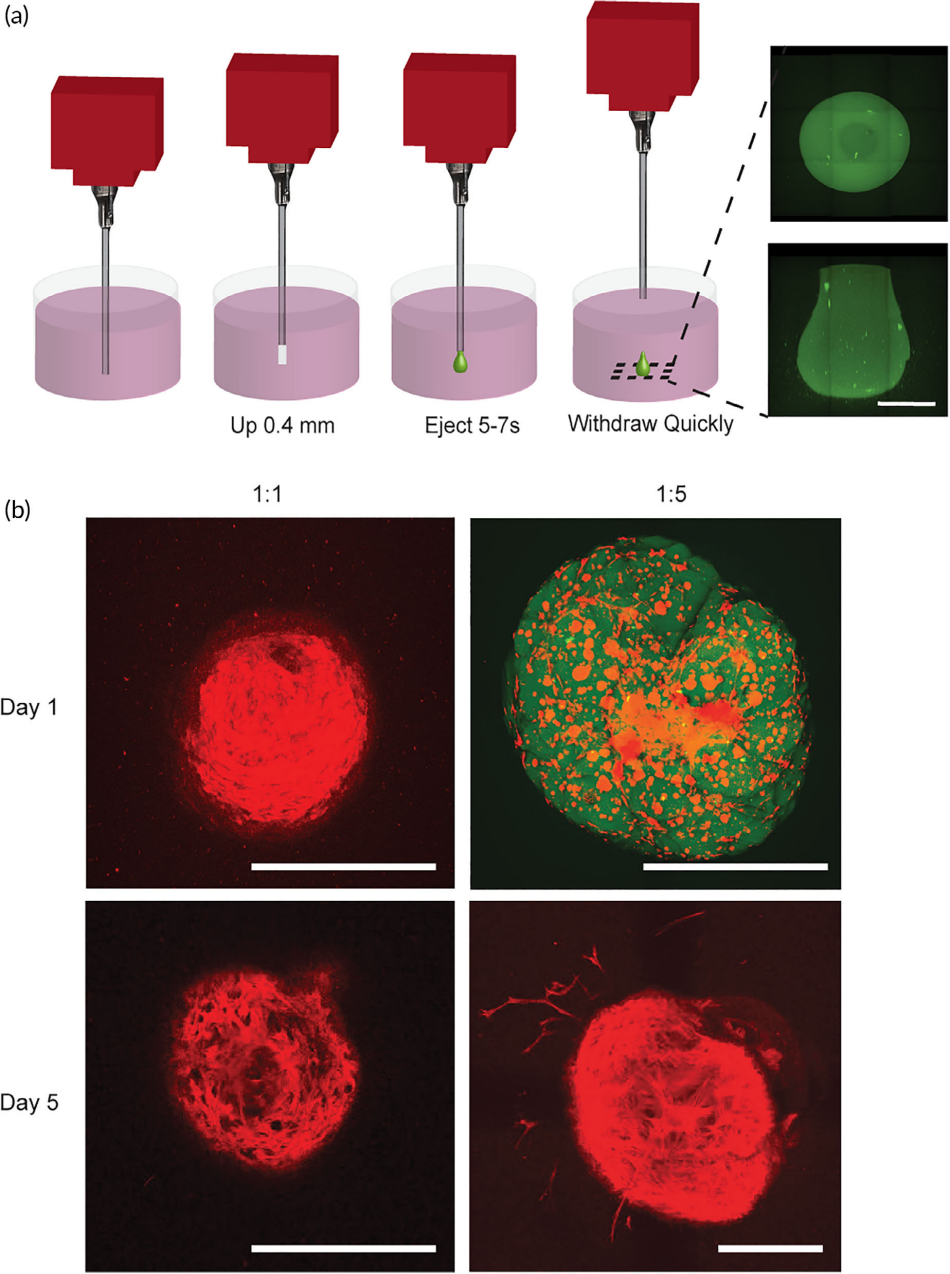
Cell administration studies *in vitro*. (a) Schematic explaining experimental set‐up of *in vitro* hydrogel injection experiments. 3D bioprinter is injected in 2.5 mg/ml collagen hydrogel, raised up 0.4 mm, hydrogel is ejected for 5–7 s from the nozzle, and removed quickly out of the collagen hydrogel. Images shown on the right are of a FITC‐labeled 1:5 PNP hydrogel from below (top image) and a sideview (bottom image). Scale bar represents 500 μm. (b) Maximum intensity confocal images of hMSCs encapsulated and delivered in 1:1 and 1:5 PNP hydrogels into collagen hydrogel across a 500 μm z‐stack. Cellular actin is stained with TRITC phalloidin (red) and the HPMC‐C_12_ is modified with 1 wt.% FITC (green). Images are from below. All scale bars represent 500 μm

Upon administration, therapeutic cells must integrate with tissue at the injection location, by attaching and migrating into the native tissue. The cell delivery community lacks biomimetic techniques for understanding the entire cell delivery process *in vitro*. We developed a novel *in vitro* experimental procedure that closely follows the *in vivo* cell delivery process. A small injection (around 15 μl) of dye‐labeled hMSC‐loaded PNP hydrogel was injected into tissue mimetic soft collagen hydrogels (2.5 mg/ml) using a 3D bioprinter (Figure 5a). The PNP hydrogel was labeled with a dye by modifying the HPMC‐C_12_ polymer with FITC prior to hydrogel fabrication.

Following administration into the tissue mimic, both the PNP hydrogel and the encapsulated hMSCs were monitored over the course of 5 days (Figure 5b) with fluorescence confocal imaging. On Day 1, the 1:5 PNP hydrogel remained intact with cells completely suspended in the administered gel. In contrast, the 1:1 PNP hydrogel had already dissolved (i.e., dye‐labeled hydrogel was no longer present) and hMSCs had completed settled into a dense layer at the bottom of the injection site. On Day 5, the 1:5 PNP hydrogel had dissolved, but hMSCs had uniformly migrated out of the injection site into the surrounding collagen hydrogel tissue mimic. These results indicate that a uniform cell suspension and more robust hydrogel structure with slower dissolution is required to improve the uniformity of cell delivery and enhance tissue integration. See Figure S2 for more replicates of representative images.

### 2.6 PNP hydrogels enhance cell retention and Immunoprotection in vivo

**Figure 6:**
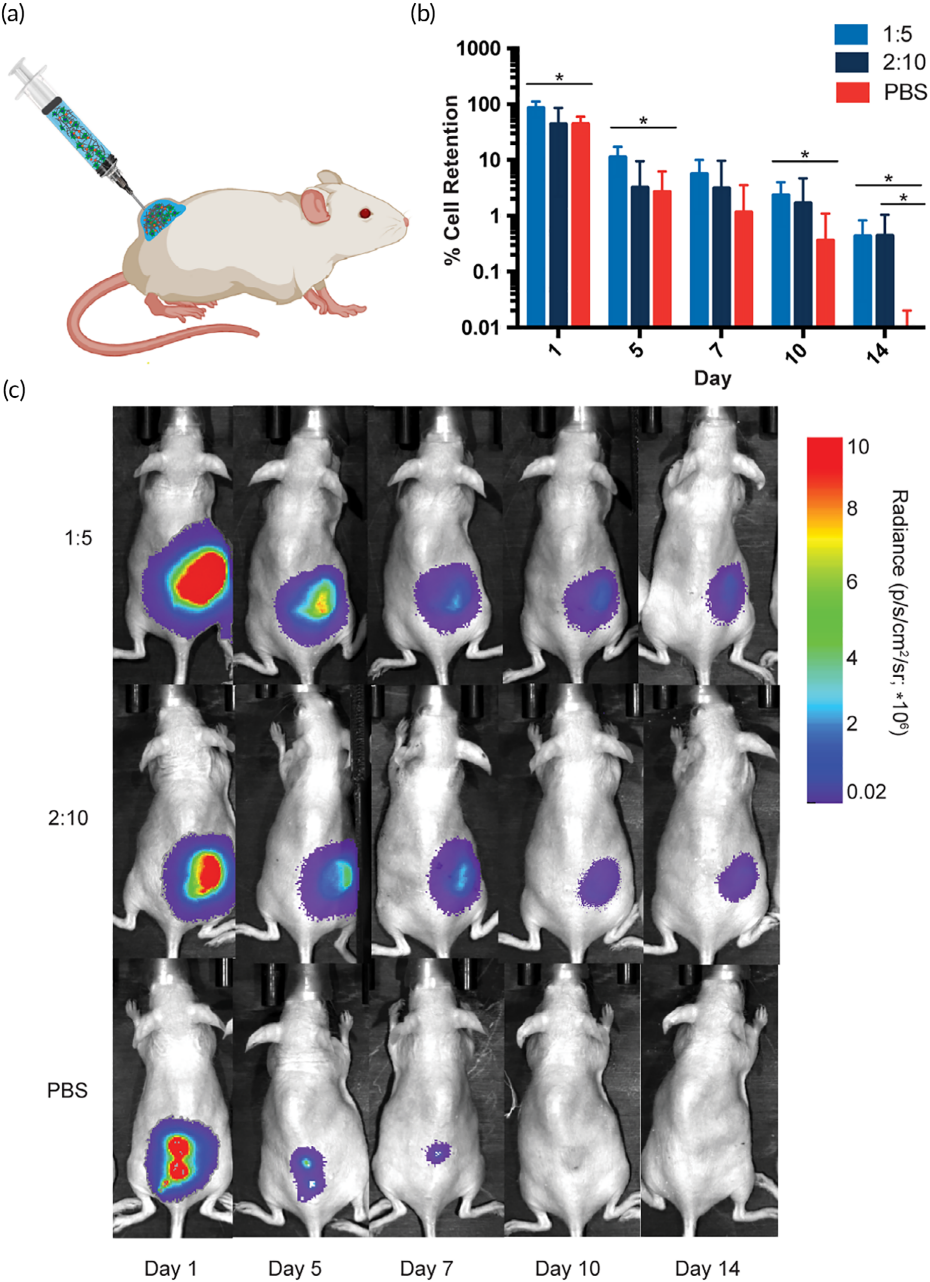
Cell retention experiments in immunocompetent SKH1E mice. (a) Schematic of *in vivo* cell retention experiment. hMSCs are delivered in PNP hydrogels into SKH1E mice in the subcutaneous space. Reporter probe potassium D‐luciferin is injected into the subcutaneous space prior to imaging. (b) % cell retention based on the total flux of photons/s in the region of interest relative to Day 0 (data shown as mean ± *SD*; *n* = 5). (c) Representative images demonstrating cell retention in 1:5 PNP, 2:10 PNP and PBS across 2 weeks

To understand the utility of the PNP hydrogel system for cell delivery *in vivo*, hMSCs were transduced with D‐luciferase to track local cell retention at the injection site. Luc^+^ hMSCs were encapsulated in PNP hydrogels and injected subcutaneously in SKH1E mice, a fully immunocompetent strain of mice (Figure 6a). Cell retention at the injection site in hydrogels with varying mechanical properties was monitored for 2 weeks with an *in vivo* Imaging System (IVIS), and the cell population was quantified as the bioluminescent signal relative to Day 0 (Figure 6b,c). To simultaneously evaluate the potential for PNP hydrogels to provide immunoprotection, we investigated hMSC administration in PNP hydrogels in both immunocompetent mice and athymic nude mice that lack an adaptive immune system (Figure S3). Previous hydrogel cell retention studies have solely studied cell retention in athymic mice.

In immunocompetent mice, the 1:5 PNP hydrogel significantly increased cell retention compared to a PBS liquid injection over the course of 2 weeks (Figure 6b,c). By Day 14, the hMSCs delivered in PNP hydrogels exhibited a 40‐fold greater relative bioluminescent signal compared to the PBS control. While the 2:10 PNP hydrogel exhibits stronger mechanical properties, it performed with higher variability in cell retention throughout these experiments than the 1:5 PNP hydrogel formulation. It is possible that the 2:10 PNP hydrogel network structure limits diffusion of nutrients and thus reduces cell viability, suggesting there may exist a tradeoff between stronger mechanical properties that enhance persistence and limited diffusion through the characteristic mesh of the polymer network. We observed greater performance enhancement with the 1:5 PNP when comparing hMSC retention experiments in immunocompetent mice than in athymic mice (Figure S2), indicating PNP hydrogels may exhibit immunomodulatory effects that provide greater protection of the implanted cells.

## 3 CONCLUSION

hMSCs are a highly clinically relevant cell type that express therapeutic paracrine signaling molecules, but researchers are still optimizing delivery methods to retain hMSCs at specific injection sites. We report a novel injectable PNP hydrogel for enhanced delivery and prolonged local retention of hMSCs. This hydrogel system utilizes dynamic multivalent interactions between hydrophobically‐modified cellulose derivatives and the surfaces of nanoparticles. The dynamic nature of these physical crosslinking interactions makes these hydrogels highly shear‐thinning and enables facile injection, complete self‐healing immediately after injection, and the formation of robust materials that can persist for long time frames in the body after injection. By modifying the PNP hydrogel structure with RGD, hMSC viability was enhanced. We demonstrate that PNP hydrogels exhibiting a yield stress can prevent cell settling across translationally relevant timescales and aid in integration with native tissue. Through these studies, we have uncovered novel experimental methods for characterization of new materials for cell delivery. Finally, we demonstrate that these PNP hydrogel materials prolong hMSC retention *in vivo* for up to 2 weeks in immunocompetent mice. PNP hydrogels present an exciting opportunity for clinical applications. In the future, the prolonged local retention of soluble factors within the PNP hydrogel in combination with therapeutic cells can be pursued to steer the fate of both the delivered cells and the surrounding tissues to increase efficacy of treatments.

## 4 MATERIALS AND METHODS

### 4.1 PEG–PLA synthesis

PEG (0.25 g, 4.1 mmol) and DBU (10.6 mg, 10 ml, 1.0 mol.% relative to LA) were dissolved in dichloromethane (DCM; 1.0 ml). LA (1.0 g, 6.9 mmol) was dissolved in DCM (3.5 ml) with mild heating. The LA solution was then added rapidly to the PEG/DBU solution and was allowed to stir rapidly for 10 min. The PEG‐b‐PLA copolymer was then recovered from the reaction medium by precipitation from excess 50:50 mixture cold diethyl ether and hexanes, collected by filtration, and dried under vacuum to yield a white amorphous polymer. DMF GPC: Mn (PDI) = 26.6 kDa (1.17).

### 4.2 HPMC‐C_12_ conjugation

HPMC (1.0 g) was dissolved in N‐methylpyrrolidone (NMP; 45 ml) by stirring at 80°C for 1 hr. Once the polymer had completely dissolved, the solution was cooled to room temperature. A solution of 1‐dodecylisocyanate (0.5 mmol) was dissolved in NMP (5 ml) and added to the reaction mixture followed by three drops of NN‐diisopropylethylamine as a catalyst. The solution was then stirred at room temperature for 16 hr. This solution was then precipitated from acetone and the hydrophobically‐modified HPMC polymer was recovered by filtration, dried under vacuum at room temperature for 24 hr and weighed, yielding HPMC‐C_12_ as a white amorphous powder.

### 4.3 RGD‐functionalized PEG–PLA synthesis

A 20 ml scintillation vial was charged with propargyl‐functional cGGGRGDSP (22.7 mg, 26.6 μmol), azido‐functional PEG–PLA (0.530 g, 21.2 μmol), and NMP (4 ml). The reaction mixture was sparged with nitrogen for 10 min, then a degassed solution (0.1 ml) containing CuBr (3.7 mg/ml) and THPTA (16 mg/ml) was added and the reaction mixture was sparged with nitrogen for a further 10 min. The reaction mixture was incubated for 16 hr at room temperature, then the polymer was precipitated from an excess of cold diethyl ether in a 50 ml centrifuge tube and recovered by centrifugation. The polymer was then dissolved in acetone and precipitated again into diethyl ether, recovered by filtration, and dried in vacuum. See Figure S4 for ^1^H‐NMR characterization.

### 4.4 PEG–PLA NP preparation by nanoprecipitation

A solution (1 ml) of PEG–PLA in a 50:50 mixture of acetonitrile and DMSO (50 mg/ml) was added dropwise to water (10 ml) at a stir rate of 600 rpm. NPs were purified by ultracentrifugation over a filter (molecular weight cut‐off of 10 kDa; Millipore Amicon Ultra‐15) followed by resuspension in water to a final concentration of 250 mg/ml. NP size and dispersity were characterized by DLS (diameter = 35 nm, PDI = 0.05).

### 4.5 hMSC culture

hMSCs (Texas A&M College of Medicine, Institute of Regenerative Medicine Cell Distribution Center) were cultured in alpha‐MEM media with 10% FBS, 1% penicillin streptomycin antibiotic, and 1% L‐glutamine. Cells were seeded at 5,000 cells/cm^2^ and media was changed every other day. Cells were split by ratio of 1:4 at each passage.

### 4.6 Cell encapsulation and hydrogel preparation

HPMC‐C_12_ was dissolved in hMSC media at 6 wt.% and loaded into a 1 ml luer‐lock syringe. A cell pellet containing the number of cells to reach the desired concentration in the final hydrogels was suspended in media. A 20 wt.% nanoparticle solution in PBS was then added to the cell suspension. The hMSC/nanoparticle solution was loaded into a 1 ml luer‐lock syringe. The cell/nanoparticle syringe was then connected to a female–female mixing elbow and the solution was moved into the elbow until it was visible through the other end of the elbow. The syringe containing the HPMC‐C_12_ polymer was then attached to the elbow other end of the elbow. The two solutions were then mixed gently back and forth through the elbow for 30 s to 1 min until the solutions had completely mixed and formed a homogenous cell‐loaded PNP hydrogel.

### 4.7 Rheological characterization

Rheology measurements were performed on a TA Instruments DHR‐2 rheometer using a 20 mm plate geometry with a gap of 750 μm. Flow sweeps were performed at shear rates from 0.01 to 100 s^−1^. Amplitude sweeps were performed at a frequency of 10 rad/s. Self‐healing step‐shear experiments were performed by switching between a shear rate of 0.1 and 10 s^−1^ every 30 s. Frequency sweeps were performed at a constant torque of 2 μNm (1.27 Pa) from 0.1 to 100 rad/s.

### 4.8 Cell viability in PNP hydrogels

Cells were encapsulated in PNP hydrogels at a concentration of 3 × 10^6^ cells/ml. Cell‐loaded PNP hydrogel (150 μl) was injected through a 26 G needle into the bottom of the wells of a 96 well plate. The plates were centrifuged mildly for 2 min to uniformly spread the gel across the bottom of the plate. After 1 and 6 days, calcein was deposited on the hydrogel and the samples were incubated for 30 min. The hydrogels were then imaged across a z‐stack of 150 μm. Maximum intensity images across the z‐stack were created using FIJI image software.

### 4.9 Cell settling experiments

hMSCs were stained with calcein dye and encapsulated in PNP hydrogels at 5 × 10^6^ cells/ml. hMSC‐loaded hydrogel (100 μl) was injected into a cuvette. The cuvette was imaged on its side in a confocal microscope through a 100 μm z‐stack. The cuvette was then placed upright for predetermined amounts of time, and then imaged on its side to assess cell settling. Maximum intensity images across the z‐stack were created using FIJI image software.

### 4.10 *In vitro* cell injection experiments

Collagen gels (2.5 mg/ml) were made in the wells of a 24 well plate according to manufacturer's protocols. In brief, neutralized collagen solution (750 μl) was deposited in each well and allowed to gel for 1 hr at 37°C. hMSCs encapsulated in PNP at 8 × 10^6^ cells/ml were loaded into a syringe with a blunt‐end 26 G needle. For these studies, the HPMC‐C_12_ polymer was labeled with FITC via isocyanate conjugation. A 3D bioprinter was used to puncture the collagen gel so that the syringe needle end was 0.5 mm from the bottom of the plate. The needle was raised 0.4 mm to create space for the hydrogel to fill upon extrusion. Approximately 15 μl of PNP was deposited into the collagen gel. Following deposition of the material, the needle rested in the collagen gel for 30 s to allow for complete pressure equilibration. The needle was quickly removed from the collagen gel. The cell‐loaded plates were then incubated at physiological temperature. On Days 1 and 5, the cells were fixed and stained with TRITC phalloidin. Cells were imaged across a z‐stack of 500 μm with 10 μm increments. Maximum intensity projections were generated in FIJI image software. See Figure S2 for more replicates of representative images.

### 4.11 hMSC luciferase transduction

Passage 1 hMSCs were plated at 3,000 cells/cm^2^ on 9 cm^2^ retronectin‐coated plates. FGF (15 μg/ml) was supplemented to the hMSC media throughout and after the transduction. After 1 day of culture, Qiagen luciferase positive transduction microparticles were deposited on the cells at a multiplicity of infection of 40. The cells were incubated with microparticles for 30 hr. The cells were cultured for 3 days and split once before placing the selective antibiotic, puromycin, on the cells at a concentration of 1 μg/ml. The Luc^+^ hMSCs were then cultured according to standard protocols outlined above.

### 4.12 *In vivo* cell retention experiments

All experiments followed protocols approved by the Stanford Administrative Panel on Laboratory Animal Care. Treatments (200 μl) were administered via subcutaneous injection onto the flank of either SK1E mice (Charles River #447) or athymic nude mice (Charles River #490). Transduced Luc^+^ hMSCs were encapsulated in PNP gels or PBS at either 2.5 × 10^6^ cells/ml (SKH1E) or 8.5 × 10^5^ cells/ml (athymic). To visualize cell retention, mice were subcutaneously injected with 100 μl of reporter probe potassium D‐luciferin at 15 mg/ml. Mice were anesthetized with isoflurane gas and imaged with an exposure time of 60 s every 5 min using an *in vivo* Imaging System (IVIS Lago) until the peak bioluminescent signal was reached for each treatment. Signal was quantified as the total flux of photons/s in the region of interest at peak intensity. Cell retention was defined as the bioluminescent signal relative to Day 0.

### 4.13 Statistical analyses

All data are reported as the mean with error bars representing *SD*. Data are classified as significant if *p* < .05 as determined by a Student's *t* test.

## Supporting information


**Data S1** Supporting information Figures S1–S4.
