# Supplementary material for: Injectable supramolecular polymer–nanoparticle hydrogels enhance human mesenchymal stem cell delivery
Source: Bioeng Transl Med. 2019 Oct 22;5(1):e10147. doi: 10.1002/btm2.10147 (PMC6971438; doi:10.1002/btm2.10147)
Supplement: Supplementary file 1 — Data S1 Supporting information Figures S1–S4. [file BTM2-5-e10147-s001.pdf]

Supplementary Information

Injectable Supramolecular Polymer-Nanoparticle Hydrogels Enhance Human Mesenchymal Stem Cell Delivery

Abigail K. Grosskopf[1], Gillie A, Roth[2], Anton A. Smith[3], Emily C. Gale[4], Hector Lopez Hernandez[3], Eric A. Appel[3,]*

1. Department of Chemical Engineering, Stanford University, Stanford, CA 94305
2. Department of Bioengineering, Stanford University, Stanford, CA 94305
3. Department of Materials Science & Engineering, Stanford University, Stanford, CA 94305
4. Department of Biochemistry, Stanford University, Stanford, CA 94305
** Denotes Corresponding Authorship: eappel@stanford.edu*

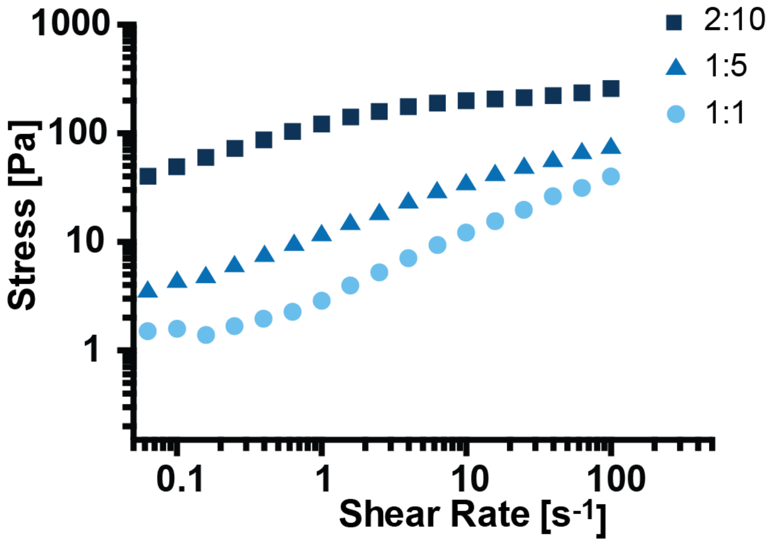

Figure S1: Steady shear rate sweeps from low to high shear rate of various PNP formulations. Stress as a function of shear rate.

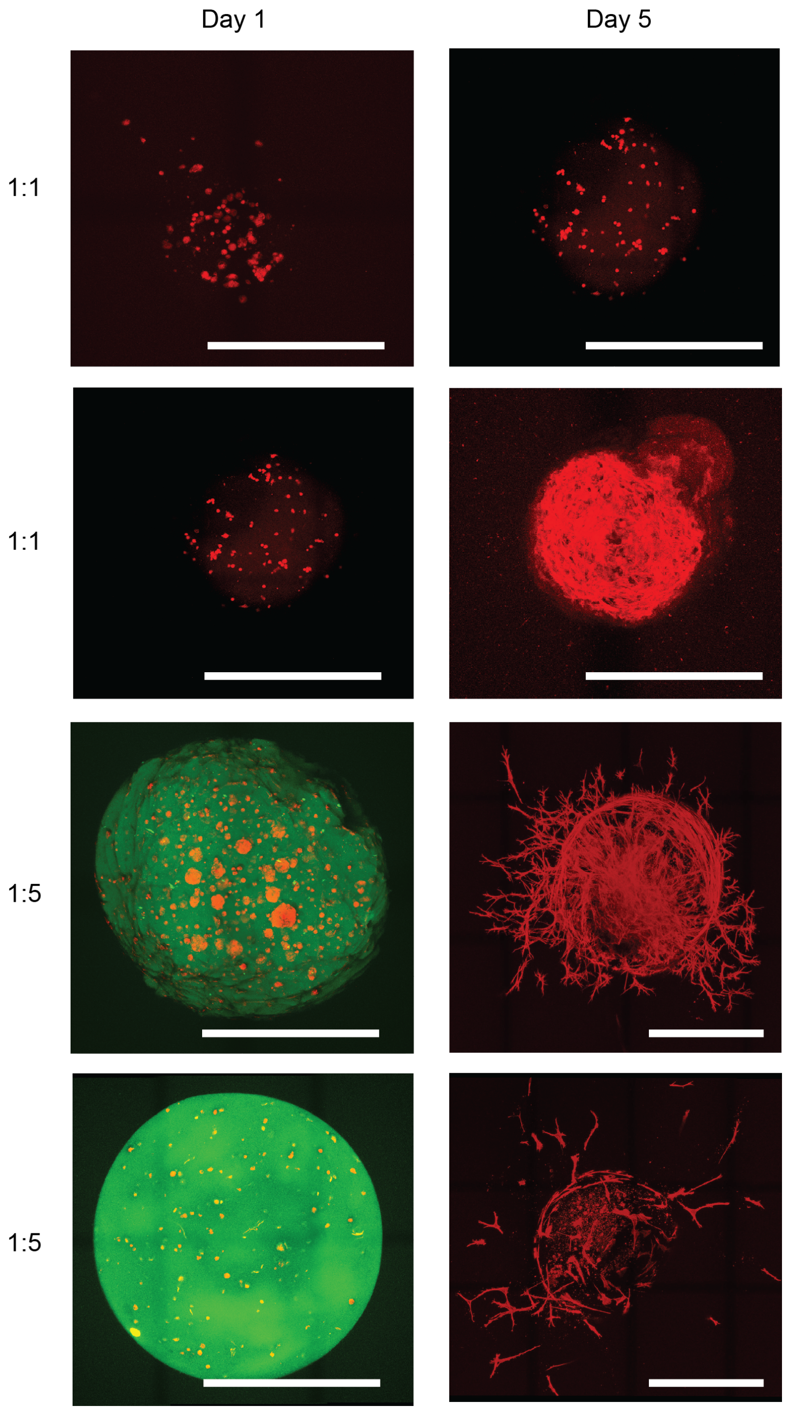

Figure S2: Maximum intensity confocal images of hMSCs encapsulated and delivered in 1:1 and 1:5 PNP hydrogels into collagen hydrogels across a 500 μm z-stack. Cellular actin is stained with TRITC phalloidin (red) and the HPMC-C_{12} is modified with 1 wt% FITC (green). Images are from below. All scale bars represents 500 μm.

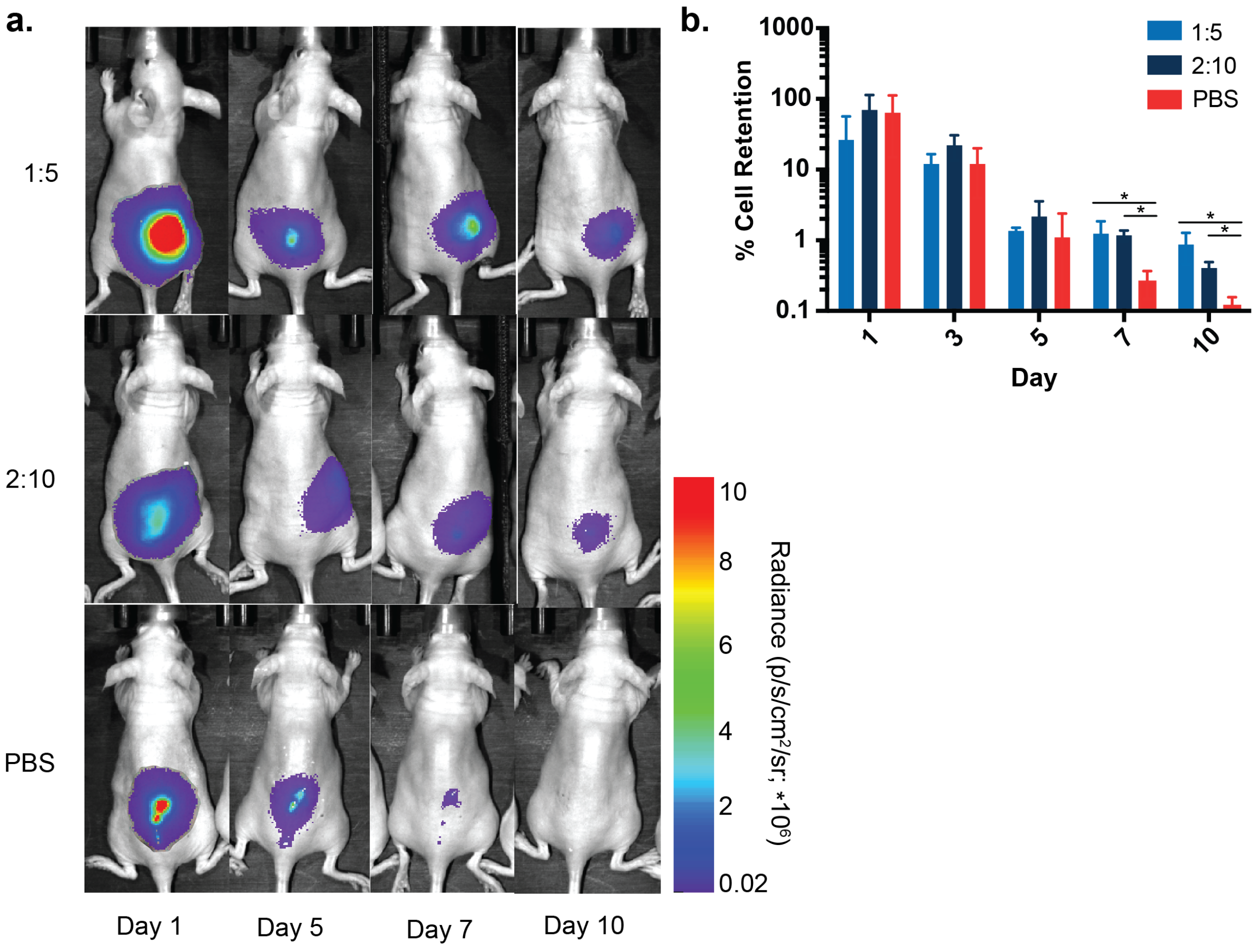

Figure S3: a. Representative images of hMSC cell retention in athymic nude mice across 10 days in PNP formulations and a bolus control. **b.** Proportion of initial cells retained locally over time (data shown as mean ± s.d.; n=3).

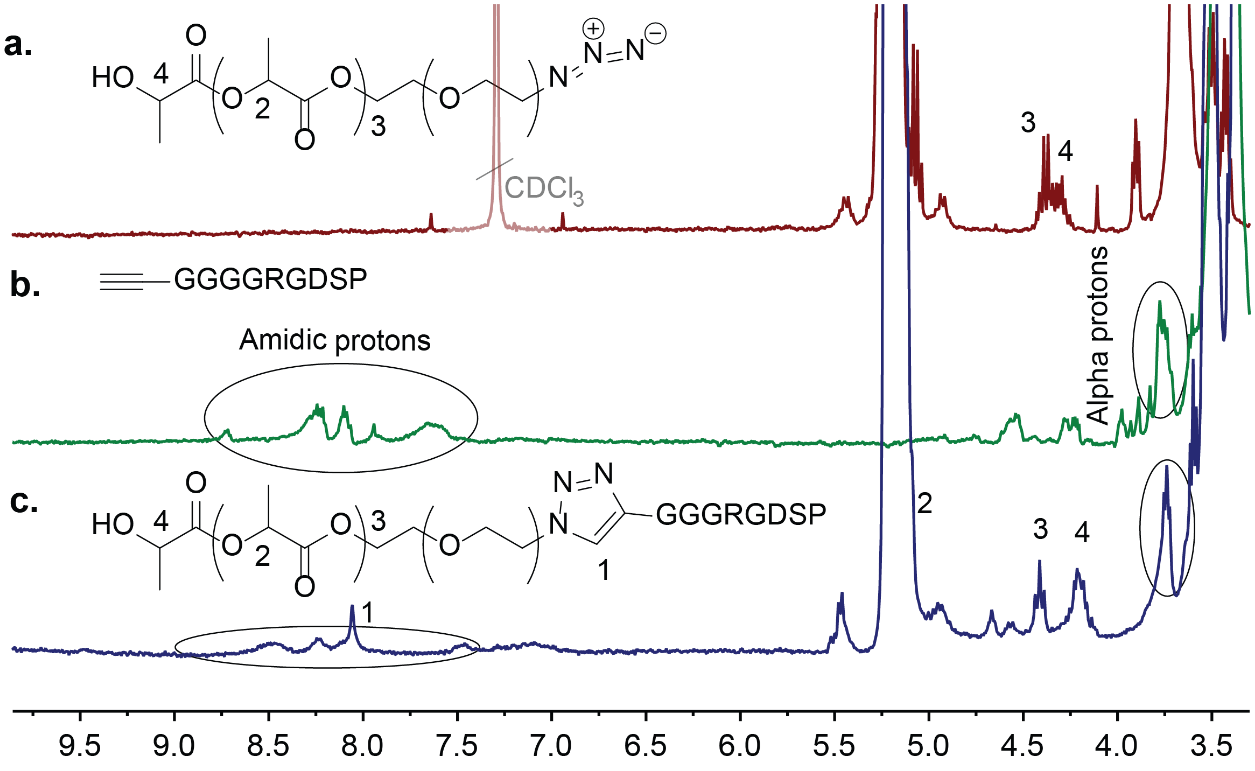

Figure S4: **a.** ^{1}H-NMR ($CDCl_3$) of N_3-PEG-PLA. **b.** ^{1}H-NMR (DMSO-*d*6) of propargylglycine-GGGRGDSP. **c.** ^{1}H-NMR (DMSO-*d*6) of RGD-PEG-PLA copolymer showing the emergence of the triazole proton at 8.05 ppm, indicating coupling of the RGD moiety to the PEG-PLA polymer.
